# Avian Diversity in the Ethiopian Orthodox Churches and Monasteries in the Case of Jer Silase Monastery in North Shoa Zone, Ethiopia

**DOI:** 10.1155/tswj/2958149

**Published:** 2025-11-03

**Authors:** Tamenut Desalegn, Chalachew Alemneh, Guta Diriba, Geleta Shasho

**Affiliations:** Department of Wildlife and Ecotourism Management, Wolkite University, Wolkite, Ethiopia

**Keywords:** avian species, distribution, habitat preference, monastery, Orthodox church, relative abundance, sanctuaries, species composition, species richness

## Abstract

Ethiopian Orthodox churches and monasteries help as critical biodiversity sanctuaries, mainly for bird species. The study was carried out in and around Jer Silase Monastery between October and January of 2021 using a stratified sample design by dividing the study area into four habitat types: riverine, cliff, natural forest, and farmland. Both the transect and point count method techniques were employed. A total of nine transects and 96-point stations methodically created. Of the transects, five were applied in the cliff habitat and four were in the riverine. Of the point stations, 51 were applied in the natural forest and 45 were applied at the farmland. The data was analyzed using Mann–Whitney and Kruskal–Wallis statistical tests. A total of 116 species, belonging to 16 orders and 49 families, were recorded during the study period. Four species, namely, Abyssinian catbird, Abyssinian woodpecker, Harwood's francolin, and yellow-fronted parrot, are unique to Ethiopia, while the scavengers white-backed vultures and hooded vultures are critically endangered species. In all four habitat categories, there were significant differences in the mean abundance of bird species (*p* = 0.001). There was no visible variation in bird species abundance between the wet and dry seasons (*p* = 0.085). During the dry season, the cliff had the lowest diversity index (*H*′ = 2.33), while the natural forest had the highest diversity (*H*′ = 4.24). The natural forest had the maximum diversity (*H*′ = 4.16) during the rainy season, whereas the cliff habitat had the lowest diversity (*H*′ = 2.94). During the dry season, the highest evenness (*J* = 0.85) was recorded at the riverine, and the least evenness (*J* = 0.39) was recorded at the cliff. Also, during the wet season, the highest evenness (*J* = 0.72) was recorded in the natural forest, and the least (*J* = 0.57) was recorded at the cliff. Enhancing community-based protection and integration traditional spiritual values with contemporary conservation strategies is essential for safeguarding bird species and their habitats in the current study area as well as in other Ethiopian monasteries.

## 1. Introduction

Different researches supported that the Ethiopian Orthodox Tewahedo Church (EOTC) is a sanctuary of biodiversity [1] and habitat for endemic species [2, 3]. The doctrine of a church positively interacts between human development and the environment. It has a long history of afforesting, preserving, and protecting indigenous trees in its sites and helping as a center of biodiversity [4–6]. Most of the areas nearby the church are covered by dense forests, and they are sanctuaries for endangered biodiversity including animal species ranging from small microbes to large animals [7]. Additionally, EOTCs and monasteries, often constructed on hills and mountains, preserve densely forested areas that provide housing and endorse biodiversity [8]. These sacred sites harbor higher flora and fauna diversity compared to nearby unprotected areas [9].

The Ethiopian Orthodox churches and monasteries, represented by sites like Jer Silase Monastery, are critical biodiversity sanctuaries, mainly for bird species, including endemic species due to their conserved forest ecologies and holy preservation practices [10, 11]. Some bird species are restricted to a few areas, highlighting the importance of these sanctuaries [12]. The larger and older church forests are mainly effective in endorsing bird species richness, diversity, and flexibility [11]. Additionally, small forest patches can exclusively support high plant biodiversity, which has a crucial role in maintaining diverse birds [1]. The richness and diversity of bird species are vital bioindicators of ecosystem health [7].

Despite their importance, church forests face several challenges, including fuel wood collection, charcoal production, and timber production [10, 13]. Due to the community's dependency on the church forest for their daily needs, for instance, fuel wood collection, indigenous tree species are extinct from surrounding environments [8, 14]. As a result of this disturbance of habitat, it affects birds' diversity and abundance [15].

Previous studies revealed Ethiopia has been blessed with 822 bird species, 18 of which are endemic [16] and 39 are threatened globally [17], but studies on birds have neglected their variety and distribution, especially in monastery forests. The checklist of birds in different monasteries remains incomplete; however, the monasteries are important habitats for different avian species [3]. In Jer Silase Monastery, there is limited scientific documentation and understanding of the biodiversity supported by these religious sites' strategies. Quantifying bird species richness and diversity is an essential step toward developing effective conservation strategies [10]. Further research is needed to explore information and the importance of such religious sanctuaries in ecological conservation and inform sustainable strategies [9]. There has been no bird research done in the Jer Silase Monastery and its surrounding areas. To date, studies devoted to the distribution and diversity of the species of birds in the Jer Silase Monastery and its surrounding areas have been lacking. To address the information gap regarding the diversity and distribution of bird species in the study area, this study attempts to gather baseline data on avifaunal diversity within Jer Silase Monastery and surrounding church forests.

## 2. Materials and Methods

### 2.1. Study Area Description

**Figure 1:**
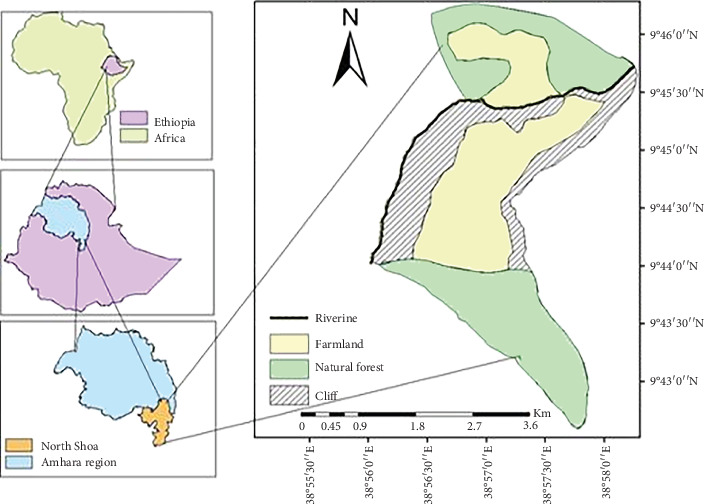
Map of the study area.

The study area, Jer Silase Monastery, is situated in northern Ethiopia. The monastery is 135 km north of Ethiopia's capital, Addis Ababa [12]. It is located between latitudes 38°57⁣′07⁣^″^ E and 9°44⁣′80⁣^″^ N (Figure 1). Its altitude ranges from 2100 to 2551 m above sea level [18]. With valleys and small mountain ridges, the area is a huge escarpment of hills and plateaus [12]. Its rainfall patterns are unimodal. Rainfall peaks in July and August and occurs primarily during the four months from June to September. There is between 800 and 1200 mm of precipitation annually. In the study area, the mean monthly maximum and minimum temperatures are 23.5°C and 16°C, respectively.

#### 2.1.1. Flora and Fauna

The research area is abundant in diverse wildlife and indigenous plant species. In and around Jer Silase Monastery, there are over a hundred different plant species [12], such as sticky sumac (*Rhus copallin*), olive tree (*Olea africana*), the African buckthorn (*Rhamnus prinoides*), Bushman's tree (*Buddleia polystachya*), bush plum (*Carpobrotus edulis*), African wormwood (*Combretum collinum*), the African wild olive (*Cordia africana*), the Ethiopian croton (*Croton macrostachyus*), sticky hopbush (*Dodonaea viscosa*), the pink dombeya (*Dombeya goetzenii*), bitter guava (*Euclea racemosa*), prickly pear cactus (*Ficus carica*), African fig (*Ficus capensis*), hairy grewia (*Grewia ferruginea*), African boxwood (*Maytenus arbutifolia*), wax myrtle (*Myrica salicifolia*), African pokeweed (*Phytolacca dodecandra*), Schimper's premna (*Premna schimperi*), natal sumac (*Rhus natalensis*), African sumac (*Rhus retinorrhoea*), African giant-rat bean (*Entada abyssinica*), and candelabra tree (*Euphorbia candelabrum*). Presenting these indigenous plant species in the study area is suitable for nesting, roosting, shelter, and feeding sites for birds.

In addition, the main mammalian species in the study area are the gelada baboon (*Theropithecus gelada*), olive baboon (*Papio anubis*), vervet monkey (*Chlorocebus pygerythrus*), rock hyrax (*Procavia capensis*), spotted hyena (*Crocuta crocuta*), honey badger (*Mellivora*), porcupine (*Hystrix cristata*), common jackal (*Canis aureus*), and Abyssinian hare (*Lepus habessinicus*) [12]. The study area having these mammal species initiated further study because mammals are one of the indicators of a good habitat.

### 2.2. Sampling Design and Data Collection

Prior to the actual data collection, reconnaissance surveys were carried out in December 2021 to formulate sampling plans and gather fundamental information regarding the study site, which encompassed accessibility, climate, topography, infrastructure, fauna, and species distribution [9, 10, 12, 19]. Furthermore, these surveys were aimed at identifying roosting and nesting locations for birds within the study area [20]. Based on the preliminary evaluation, a stratified random sample design was employed. Four types of habitats were identified in the area: farmland (cultivated area), cliff habitat, natural forest, and the riverine. Overhangs, shelter caves, and shelters in rock are characteristics of the rocky and cliff ecosystems. Dispersed trees, areas cleared for agriculture, and populated areas are examples of habitats that have been altered by humans. The naturally occurring forest is made up of native or naturally dispersed tree species and strains that reproduce themselves [12]. Riverine is a natural stream of water flowing in a definite course or channel or series of diverging and converging channels during the wet season, fairly large in size due to flooding.

The study took into consideration the dry and wet seasons from January to October 2021. From this time interval, in total, the data was taken for 10 months: five months for the wet season and five months for the dry season. The months of January through May are considered the dry season, while June through October is considered the wet season [12]. Both line transect and point count techniques were employed based on the habitat type [21]. Line transects are useful in open environments, such as farmlands, cliffs, and riverine; the point count method used in the natural forest is a result of the inaccessibility of using line transects [22]. At the cliff habitat, the line transects were designed at the base and aside from the cliff because it is difficult to design all parts of the cliff.

Using ArcGIS V 10.8.1, a total of nine sampling line transects and 96-point count stations were methodically created by habitat types or habitat conditions [23]. Of them, five were used in the cliff habitat and four were used in the riverine based on the area coverage of the habitat type [24]. The transect length measured varied from 1 to 1.1 km due to habitat variation. The area coverage of the study habitats includes 60 ha of riverine, 80 ha of cliff, 108 ha of farmland, and 106 ha of forest. The spacing between each sampling transect was 250 m to prevent the counting of bird species twice [10]. Depending on the weather and the time of day, the data were collected from 6:30 a.m. to 10:00 a.m. in the morning and 3:00 p.m. to 6:00 p.m. in the afternoon when the birds were active in performing multiple activities [25, 26]. The data were collected three times per week, a total of 12 times every month. In total, 120 sampling days were taken in both dry and wet seasons, 60 in the wet season and 60 in the dry season. Sampling areas were located using Geospatial Positioning System (GPS 72 Hz) to ensure the accuracy of the data. We traversed the transects and point stations, identifying bird species with the naked eye and using field binoculars to enhance visibility at a distance [27]. We recorded the sightings in a notebook before entering the data into SPSS using a pen to maintain a checklist of birds and a bird guidebook for identification assistance [28]. In the point station survey, the observation process began with standing silently in the center to reduce disturbances [10]. This initial phase permitted birds to settle, with the observer remaining still for 2–5 min. Subsequent to this period of silence, the observer directed a 360° rotation to record the bird species present. Moreover, for the line transect, the observer walked along the center of the line, recording bird species observed on both the right and left sides. Data collectors worked simultaneously across the transects and made necessary adjustments. To differentiate between species, they utilized features such as external morphology (form, color, size, beak, legs, and tail), song, call, plumage patterns, and habitat type [22, 29].

### 2.3. Data Analysis

The data were compiled into an Excel spreadsheet according to the seasons for each type of habitat. The statistical analysis was conducted using the statistics package SPSS Version 23.0 [30]. The Shannon–Wiener diversity (*H*′) and evenness (*J*/*E*) were computed using PAST (Paleontological Statistics) [31]. The impacts of season on species abundance and population variations among habitats were examined by Mann–Whitney and Kruskal–Wallis statistical tests, respectively.

To compare estimated (based on rarefaction and extrapolation) species richness among treatments (habitat types or seasons), we computed estimated species richness S(est) with 95% confidence intervals (CI). We used nonoverlapping 95% CIs of S(est) (based on extrapolation) at the reference sample size (the largest sample size among habitats) as a conservative criterion for statistical differences in species richness between habitat types, in line with other researchers [15].

The formula for calculating the relative abundance of a bird species was RA = *n*/*N*∗100, where *n* is the number of individuals of a criterion species that have been documented and *N* is the total number of individuals in the species [32]. The relative abundance value (< 0.1) indicates a rare abundance category, 0.1–2.0 indicates an uncommon abundance category, 2.1–10.0 indicates a frequent abundance category, 10.1–40 indicates a common abundance category, and > 40 indicates an abundance category [32].

## 3. Results

### 3.1. Species Composition and Richness

**Figure 2:**
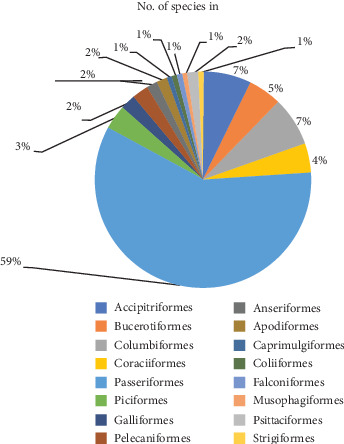
Number of species recorded from different orders in percent.

A total of 11,842 individual birds of 116 species, belonging to 16 orders and 49 families, were recorded in the study area during the study period (Appendix 1). Of these, 5206 (44%) and 6636 (56%) birds were observed during the wet and dry seasons, respectively (Table 1). Passeriformes was the most abundant order in terms of the number of families (32 families) and species (73 species). Order Bucerotiformes was the second most abundant in terms of the number of families (six families) and species (six species). On the other hand, although having nine and eight species, respectively, the two bird orders Columbiformes and Accipitriformes were represented by a single family (Figure 2). Of 116 identified bird species, Harwood's francolin (*Pternistis harwoodi*), Abyssinian catbird (*Sylvia galinieri*), Abyssinian woodpecker (*Dendropicos abyssinicus*), and yellow-fronted parrot (*Poicephalus flavifrons*) are endemic to Ethiopia. Based on the IUCN Red List category status, two species, the lammergeier (*Gypaetus barbatus*) and Harwood's francolin (*Pternistis harwoodi*), are near threatened. Hooded vulture (*Necrosyrtes monachus*) and white-backed vulture (*Gyps africanus*) are critically endangered. The African hoopoe (*Upupa africana*) is endangered, and the tawny eagle (*Aquila rapax*) is vulnerable. The remaining 110 species of birds are of least concern.

**Figure 3:**
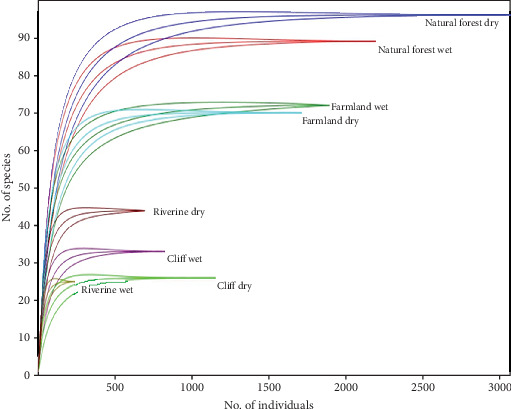
Rarefaction result of species richness of the birds in four habitats in wet and dry seasons of the four habitat types.

The mean abundance of bird species significantly varied in the three four types (Kruskal–Wallis test; *χ*^2^ = 204.8, df = 3, *p* = 0.001). Bird species richness is highest in natural forests, followed by farmland, riverine, and cliffs, which have the lowest diversity in bird species. Table 1 and Figure 3 show the variation of species richness in the four habitat types. However, bird species abundance was not significantly different across seasons (*U* = −1.72, *p* = 0.085).

### 3.2. Species Diversity and Distribution

Irrespective of habitat type, the Shannon diversity index was similar during the dry and the wet seasons (Table 2). Regarding season, the Shannon diversity index was highest in the natural forest (*H*′ = 4.2), followed by the farmland (*H*′ = 3.9), riverine (*H*′ = 3.6), and cliff (*H*′ = 2.9) habitat types (Table 2). Moreover, during the dry season, the highest evenness (*J* = 0.85) was recorded at the riverine, and the least evenness (*J* = 0.39) was recorded at the cliff. Also, during the wet season, the highest evenness (*J* = 0.72) was recorded in the natural forest, and the least (*J* = 0.57) was recorded at the cliff. The highest Simpson diversity (*D* = 0.98) was recorded in the natural forest during both the dry and the wet seasons, followed by farmland (*D* = 0.97) and riverine (*D* = 0.96). Moreover, the highest richness of bird species recorded at the natural forest is shown in Figure 3.

### 3.3. Relative Abundance

As shown in Appendix 2, based on the relative abundance category, four species were rare, 100 species were uncommon, and 12 species of birds were in the frequent category during the wet season. Moreover, during the dry season, one species was recorded as rare, 108 species were under uncommon, and seven species were in the frequent category. Hooded vultures were more abundant, accounting for 6.4% in the wet and 7.9% in the dry season; secondly, white-backed vultures accounted for 3.7% and 5.2% during the wet and dry seasons, respectively, in the cliff habitat. Moreover, yellow-crowned bishops accounted for 4.3% and 3.9% during the wet and dry seasons, respectively, in the farmland.

## 4. Discussion

The study area possesses a high number of birds, including endemic, endangered, and critically endangered species. This was probably due to the favorable habitat for nesting, breeding, feeding, and sheltering sites for bird species [33]. Additionally, the monastery contains heterogeneous vegetation cover of the habitats, and large canopy sizes probably were the ideal environment for the species to survive. Two species, the Harwood's francolin (*Pternistis harwoodi*) and the Lammergeier (*Gypaetus barbatus*), are classified as near threatened by the IUCN Red List, and the white-backed vulture (*Gyps africanus*) and hooded vulture (*Necrosyrtes monachus*) are critically endangered. Due to having these endangered species, the study area needs conservation priority [34]. Compared with the other study conducted by [10] adjacent to Debre-Libanos Monastery Forest, the number of bird species reported in the present study area has high species diversity, where they recorded 61 bird species. The study area's high bird species diversity may have been linked to variations in the vegetation's composition and characteristics associated with habitat [35]. In contrast, this study [36] at Tara Gedam Monastery Forest and adjacent habitats reveals that high bird diversity exists compared to the present study; they recorded 98 avian species. This might be due to the levels of human disturbance, such as less anthropogenic practices, that may not highly affect the bird population.

The largest recorded population and number of species belong to the order Passeriformes. A similar survey [22, 26, 37] revealed that the order Passeriformes had the greatest number of species recorded. Due to the rapid evolution of passerine species, their adaptability to all terrestrial settings, and the abundance of species, most bird species are known to exist in the order Passeriformes [38]. Next to the Passeriformes order, Bucerotiformes was the second most abundant in terms of the number of families (six families) and species (six species). On the other hand, despite having nine and eight species, respectively, the bird orders Columbiformes and Accipitriformes were occupied by a single family (Figure 2). The adaptability of every ecological setting in the study area might have been the reason behind it.

The species richness and abundance of the three habitat types in this study differ. During the dry and wet seasons, natural forests had the highest recorded bird species richness and diversity. The explanation for this could be that birds receive more food, places to roost, and nest sites, and forests can be used as overwintering and breeding habitats, which is something that is increasingly recognized during the dry season [39]. In line with this study, Desalegn and Negussie [10] in similar geographical locations revealed that a high diversity of bird species has been observed in the natural forest during both the wet and dry seasons. This is most likely because there is an adequate supply of food, nesting sites, a diverse range of flora, and diving places. According to a study backed by [35], variations in habitat features and feeding patterns were linked to variations in species diversity, species abundance, and individual numbers among various species. Furthermore, it was observed by [40] that the vegetation structure influences the distribution and abundance of numerous bird species.

Besides, during the wet season, the farming habitat displayed the highest levels of species richness and diversity next to the natural forest. A possible reason could be that, in the rainy season, a variety of crops on urban farmlands encourage the growth of weeds, which helps feed some bird species, and that various crops serve as suitable homes for tiny flies and maggots [41]. Additionally, birds walk about or perch on trellis and vine crops, which are rather frequent in farmlands with a variety of crops, and consume insects at the development of green crops. Furthermore, as per reference [42], the wet season on farmland exhibits the highest species diversity of birds because of the increased production and yield of habitats, as well as the flexibility of birds to live in environments modified by humans, leading to an increase in species richness. The third cliff habitat had higher bird richness than the riverine habitat. Cliff habitat might be used for roosting, nesting, and sunning by bird species. However, the riverine habitat had less species diversity, which might be because the poor habitat due to erosion by flood was not suitable for birds. Numerous bird species suffered as a result of these landscape alterations [43].

The distribution of species in this study did not change significantly between the wet and dry seasons. This may be related to the high levels of water, cover, and food available during the study period, all of which supported the highest levels of species richness and evenness in the ecosystem [44]. However, compared to the dry season, there was a greater diversity, number, and evenness of birds during the rainy season [26]. Between the two study seasons, there were distinguished differences in bird abundance in the study site. The effects of environmental seasonality on species distributions are significant because seasonal variations in predicted changes in the global environment are possible [45]. The highest bird abundance was seen during the wet season, while the lowest was during the dry season. This study is comparable to studies conducted by [46, 47], where the mean abundance of bird species varied significantly between the rainy and dry seasons.

In contrast to the current study [48], it reveals that a greater diversity of birds was observed during the winter than during the summer. This may be the result of birds moving more about their local area in search of food sources and of plants losing their foliage, which makes it easier for birds to be seen. Furthermore, this result supported research by [49, 50] that discovered that other species, including some raptors, migrate to better utilize their resources [51] and that more species were seen in the winter than in the summer when plants are defoliated.

Throughout the investigation, there was little variation in the relative abundance of bird species. The ordinal rank of uncommon was assigned to the majority of the bird species. This is because the diversity of the flora may cause the species to have a broad home range and high demand [10, 40]. Comparably, Genet and Ejigu [52] revealed that the Apin forest recorded a higher number of rare bird species during the wet and dry seasons. The nesting site and wide home range of bird species may be the cause of the abundance of rank uncommon bird species [10]. The two vulture species with a high relative abundance are hooded and white-backed vultures at the study site. This may be due to the fact that vultures can nest and roost in the cliff environment. According to [53], the availability of breeding, feeding, and watering sites was associated with the abundance of those species.

## 5. Conclusion

The discoveries of this study highlight the critical conservation importance of the study area, which supports an extraordinary diversity of 116 bird species, including several endemics, endangered, and critically endangered species. The presence of critically endangered species, such as the hooded vulture and white-backed vulture, emphasizes the urgency of implementing targeted conservation strategies. The observed difference in bird abundance and diversity among different habitat types—mainly the higher diversity in natural forests compared to others—suggests that habitat preservation and restoration should be prioritized. A comprehensive understanding of species distribution and habitat needs is essential for effective conservation planning in order to develop specific management strategies that take into account the constraints of surrounding anthropogenic activities as well as the needs of the birds.

To enhance conservation efforts, it is crucial to involve local communities and stakeholders in the area. Many people may lack awareness of the ecological significance of birds and the potential impacts of their activities on avian populations. Educational programs aimed at raising awareness about the importance of bird conservation and sustainable practices can foster local stewardship of natural resources. Moreover, involving communities in conservation initiatives ensures that their livelihoods are considered, reducing conflicts and promoting cooperative efforts. Given the threats posed by subsistence activities, immediate and effective management strategies must be developed to mitigate these pressures while promoting biodiversity conservation in the study area.

## Figures and Tables

**Table 1:** Mann–Whitney and Kruskal–Wallis statistical tests in season and sampling site.

**Mann–Whitney statistical test**					
**Abundance: Season**	**N**	**Mean rank**	**Sum of ranks**	**Z**	**Asymp. sig.**
Wet	123	120.18	14,782.00	−1.72	0.085
Dry	123	126.82	15,599.00		
Total	246				

**Kruskal–Wallis statistical test**					
**Abundance: Sampling site**	**N**	**Mean rank**	**χ** ^2^	**df**	**Asymp. sig.**
Natural forest	220	620.27	193.032	3	0.0001
Farmland	220	524.35			
Cliff	220	344.92			
Robi river	220	352.47			
Total	920				

**Table 2:** Bird species richness, abundance, diversity, and evenness during dry and wet seasons.

**Ecological diversity indices**	**Natural forest**	**Natural forest**	**Farmland**	**Farmland**	**Cliff**	**Cliff**	**Riverine**	**Riverine**
	**Season**		**Season**		**Season**		**Season**	
	**Wet**	**Dry**	**Wet**	**Dry**	**Wet**	**Dry**	**Wet**	**Dry**
Taxa_S	89	96	72	70	33	26	25	44
Individuals	2212	3088	1906	1727	835	1161	252	704
Dominance_D	0.02004	0.01828	0.02533	0.02384	0.09432	0.1819	0.05326	0.03115
Simpson_1-D	0.98	0.9817	0.9747	0.9762	0.9057	0.8181	0.9467	0.9688
Shannon_H	4.161	4.235	3.925	3.969	2.939	2.33	3.066	3.609
Evenness_e^H/S	0.7204	0.7192	0.7036	0.756	0.5726	0.3951	0.858	0.8389
Brillouin	4.068	4.159	3.839	3.875	2.853	2.279	2.881	3.476
Menhinick	1.892	1.728	1.649	1.684	1.142	0.7631	1.575	1.658
Margalef	11.43	11.82	9.401	9.257	4.757	3.543	4.34	6.558
Equitability_J	0.9269	0.9278	0.9178	0.9342	0.8406	0.715	0.9524	0.9536
Fisher_alpha	18.59	18.79	14.8	14.65	6.861	4.719	6.895	10.4
Berger–Parker	0.0443	0.04016	0.05876	0.06022	0.2443	0.3549	0.09524	0.06108
Chao-1	89	96	72	70	33	26	25	44

*Note: H*′, Shannon–Weiner index; J, evenness.

Abbreviation: D, dominance.

**Table 3:** Species checklist and IUCN conservation status of birds in and around Jer Silase Monastery.

**Order of species**	**Family**	**Common name**	**Scientific name**	**IUCN status**
Accipitriformes	Accipitridae	Yellow-billed kite	*Milvus aegyptius*	LC
		African fish eagle	*Icthyophaga vocifer*	LC
		Lammergeier	*Gypaetus barbatus*	NT
		Hooded vulture	*Necrosyrtes monachus*	CR
		White-backed vulture	*Gyps africanus*	CR
		Black-chested snake eagle	*Circaetus pectoralis*	LC
		African goshawk	*Accipiter tachiro*	LC
		Augur buzzard	*Buteo augur*	LC
		Tawny eagle	*Aquila rapax*	VU

Anseriformes	Anatidae	Egyptian goose	*Alopochen aegyptiaca*	LC
		Yellow-billed duck	*Anas undulata*	LC

Apodiformes	Apodidae	Little swift	*Apus affinis*	LC
		Nyanza swift	*Apus niansae*	LC

Bucerotiformes	Phoeniculidae Upupidae Bucerotidae	Black-billed wood hoopoe	*Phoeniculus somaliensis*	LC
		African hoopoe	*Upupa africana*	EN
		Red-billed hornbill	*Tockus erythrorhynchus*	LC
		Hemprich's hornbill	*Lophoceros hemprichii*	LC
		African gray hornbill	*Lophoceros nasutus*	LC
		Silvery-cheeked hornbill	*Bycanistes brevis*	LC

Caprimulgiformes	Caprimulgidae	Montane nightjar	*Caprimulgus poliocephalus*	LC

Coliiformes	Coliidae	Speckled mousebird	*Colius striatus*	LC

Columbiformes	Columbidae	Speckled pigeon	*Columba guinea*	LC
		White-collared pigeon	*Columba albitorques*	LC
		Namaqua dove	*Oena capensis*	LC
		Ring-necked dove	*Streptopelia capicola*	LC
		Red-eyed dove	*Streptopelia semitorquata*	LC
		Vinaceous dove	*Streptopelia vinacea*	LC
		Laughing dove	*Spilopelia senegalensis*	LC
		Dusky turtle dove	*Streptopelia lugens*	LC
		Lemon dove	*Columba larvata*	LC

Coraciiformes	Alcedinidae	Pied kingfisher	*Ceryle rudis*	LC
		Gray-headed kingfisher	*Halcyon leucocephala*	LC
		Giant kingfisher	*Megaceryle maxima*	LC
	Meropidae	Little bee-eater	*Merops pusillus*	LC
		Blue-breasted bee-eater	*Merops variegatus*	LC

Falconiformes	Falconidae	Lanner falcon	*Falco biarmicus*	LC

Pelecaniformes	Ardeidae	Cattle egret	*Bubulcus ibis*	LC
		Striated heron	*Butorides striata*	LC
	Scopidae	Hamerkop	*Scopus umbretta*	LC

Galliformes	Numididae	Helmeted guineafowl	*Numida meleagris*	LC
	Phasianidae	Erckel's francolin	*Pternistis erckelii*	LC
		Harwood's francolin	*Pternistis harwoodi*	NT

Musophagiformes	Musophagidae	White-cheeked turaco	*Menelikornis leucotis*	LC

Passeriformes	Alaudidae	Erlanger's lark	*Calandrella blanfordi erlangeri*	LC
		Thekla's lark	*Galerida theklae*	LC
		Rock martin	*Galerida theklae*	LC
	Hirundinidae	Lesser striped swallow	*Cecropis abyssinica*	LC
	Motacillidae	Mountain wagtail	*Motacilla clara*	LC
	Pycnonotidae	Common bulbul	*Pycnonotus barbatus*	LC
	Muscicapidae	Rüppell's robin-chat	*Cossypha semirufa*	LC
		Red-breasted wheatear	*Oenanthe bottae*	LC
		African stonechat	*Saxicola torquatus*	LC
		Abyssinian black wheatear	*Oenanthe lugubris*	LC
		Moorland chat	*Pinarochroa sordida*	LC
		Mocking cliff chat	*Thamnolaea cinnamomeiventris*	LC
		Rüppell's black chat	*Myrmecocichla melaena*	LC
		Little rock thrush	*Monticola rufocinereus*	LC
		African paradise flycatcher	*Terpsiphone viridis*	LC
		African dusky flycatcher	*Muscicapa adusta*	LC
		Abyssinian slaty flycatcher	*Melaenornis chocolatinus*	LC
	Turdidae	Groundscraper thrush	*Turdus litsitsirupa*	LC
		Mountain thrush	*Turdus plebejus*	LC
	Locustellidae	Cinnamon bracken warbler	*Bradypterus cinnamomeus*	LC
	Phylloscopidae	Brown woodland warbler	*Phylloscopus umbrovirens*	LC
	Cisticolidae	Gray-backed camaroptera	*Camaroptera brevicaudata*	LC
		Ethiopian cisticola	*Cisticola lugubris*	LC
		Tawny-flanked prinia	*Prinia subflava*	LC
	Leiothrichidae	White-rumped babbler	*Turdoides leucopygia*	LC
	Sylviidae	Abyssinian catbird	*Sylvia galinieri*	LC
	Paridae	White-backed black tit	*Melaniparus leuconotus*	LC
	Zosteropidae	Montane white-eye	*Zosterops poliogastrus*	LC
	Nectariniidae	Tacazze sunbird	*Nectarinia tacazze*	LC
		Malachite sunbird	*Nectarinia famosa*	LC
		Scarlet-chested sunbird	*Chalcomitra senegalensis*	LC
		Variable sunbird	*Cinnyris venustus*	LC
	Laniidae	Common fiscal	*Lanius collaris*	LC
		Gray-backed fiscal	*Lanius excubitoroides*	LC
	Malaconotidae	Ethiopian boubou	*Laniarius aethiopicus*	LC
	Oriolidae	Ethiopian oriole	*Oriolus monacha*	LC
	Dicruridae	Fork-tailed drongo	*Dicrurus adsimilis*	LC
	Corvidae	Pied crow	*Corvus albus*	LC
		Cape rook	*Corvus capensis*	LC
		Fan-tailed raven	*Corvus rhipidurus*	LC
		Thick-billed raven	*Corvus crassirostris*	LC
	Buphagidae	Red-billed oxpecker	*Buphagus erythrorynchus*	LC
	Sturnidae	Red-winged starling	*Onychognathus morio*	LC
		Slender-billed starling	*Onychognathus tenuirostris*	LC
		Greater blue-eared starling	*Lamprotornis chalybaeus*	LC
	Passeridae	Swainson's sparrow	*Passer swainsonii*	LC
	Ploceidae	Village weaver	*Ploceus cucullatus*	LC
		Spectacled weaver	*Ploceus ocularis*	LC
		Little weaver	*Ploceus luteolus*	LC
		Baglafecht weaver	*Ploceus baglafecht*	LC
		Yellow-crowned bishop	*Euplectes afer*	LC
		Yellow bishop	*Euplectes capensis*	LC
		Yellow-mantled widowbird	*Euplectes macroura*	LC
		Red-collared widowbird	*Euplectes ardens*	LC
	Estrildidae	Red-cheeked cordon-bleu	*Uraeginthus bengalus*	LC
		Red-billed firefinch	*Lagonosticta senegala*	LC
		African firefinch	*Lagonosticta rubricata*	LC
		Yellow-bellied waxbill	*Coccopygia quartinia*	LC
		Common waxbill	*Estrilda astrild*	LC
	Viduidae	Pin-tailed whydah	*Vidua macroura*	LC
		Village indigobird	*Vidua chalybeata*	LC
	Fringillidae	Yellow-crowned canary	*Serinus flavivertex*	LC
		African citril	*Crithagra citrinelloides*	LC
		Brown-rumped seedeater	*Crithagra tristriata*	LC
		Streaky seedeater	*Crithagra striolata*	LC
	Emberizidae	Cinnamon-breasted bunting	*Emberiza tahapisi*	LC

Piciformes	Lybiidae	Black-billed barbet	*Lybius guifsobalito*	LC
		Red-fronted tinkerbird	*Pogoniulus pusillus*	LC
	Picidae	Nubian woodpecker	*Campethera nubica*	LC
		Abyssinian woodpecker	*Dendropicos abyssinicus*	LC

Psittaciformes	Psittacidae	Yellow-fronted parrot	*Poicephalus flavifrons*	LC
	Psittaculidae	Black-winged lovebird	*Agapornis taranta*	LC

Strigiformes	Strigidae	Verreaux's eagle-owl	*Ketupa lactea*	LC

Abbreviations: CR, critically endangered; LC, least concern; NT, near threatened; VU, vulnerable.

**Table 4:** Relative abundance and distribution of bird species in the three study habitats.

**Species**	**Scientific name**	**Habitat type**								**Number of individuals**		**Number of individuals**	
		**Natural forest**		**Farmland**		**Cliff**		**Robi river**					
		**Wet**	**Dry**	**Wet**	**Dry**	**Wet**	**Dry**	**Wet**	**Dry**	**Wet**	**RA (%)**	**Dry**	**RA (%)**
Cattle egret	*Bubulcus ibis*	−	−	−	−	−	−	+	+	6	0.2	13	0.2
Striated heron	*Butorides striata*	−	−	−	−	−	−	−	+	0	0	8	0.12
Hamerkop	*Scopus umbretta*	+	+	−	−	−	−	+	+	20	0.38	33	0.5
Egyptian goose	*Alopochen aegyptiaca*	−	−	−	−	−	−	+	+	6	0.12	18	0.27
Yellow-billed duck	*Anas undulata*	−	−	−	−	−	−	+	+	7	0.13	24	0.36
Yellow-billed kite	*Milvus aegyptius*	+	+	−	−	−	−	−	−	6	0.12	4	0.06
African fish-eagle	*Icthyophaga vocifer*	−	+	+	+	+	+	−	−	24	0.46	31	0.47
Lammergeier	*Gypaetus barbatus*	−	−	−	−	+	+	−	−	6	0.12	8	0.12
Hooded vulture	*Necrosyrtes monachus*	+	−	+	+	+	+	−	−	332	6.4	528	7.96
White-backed vulture	*Gyps africanus*	+	+	+	+	+	+	−	−	194	3.7	348	5.24
Black-chested snake eagle	*Circaetus pectoralis*	+	+	−	−	+	+	−	−	12	0.23	21	0.32
African goshawk	*Accipiter tachiro*	+	+	−	−	−	−	−	−	4	0.08	8	0.12
Augur buzzard	*Buteo augur*	+	+	+	+	+	+	−	−	20	0.38	30	0.45
Tawny eagle	*Aquila rapax*	+	+	+	+	−	−	−	−	5	0.1	9	0.14
Lanner falcon	*Falco biarmicus*	−	−	+	−	+	+	−	−	18	0.35	7	0.12
Helmeted guineafowl	*Numida meleagris*	+	+	+	−	−	−	−	−	64	1.23	84	1.27
Erckel's francolin	*Pternistis erckelii*	+	+	+	+	+	+	0	+	128	2.46	190	2.86
Harwood's francolin	*Pternistis harwoodi*	+	+	+	+	−	−	−	−	22	0.42	16	0.24
Speckled pigeon	*Columba guinea*	−	−	+	+	+	+	−	+	40	0.77	75	1.13
White-collared pigeon	*Columba albitorques*	−	+	+	+	+	−	−	+	56	1.08	64	0.96
Namaqua dove	*Oena capensis*	+	+	−	−	−	−	−	−	14	0.27	41	0.62
Ring-necked dove	*Streptopelia capicola*	+	+	+	+	−	−	−	+	22	0.42	36	0.54
Red-eyed dove	*Streptopelia semitorquata*	+	+	+	+	+	−	−	−	51	0.98	57	0.86
Vinaceous dove	*Streptopelia vinacea*	+	+	−	+	−	−	−	−	14	0.27	30	0.45
Laughing dove	*Spilopelia senegalensis*	+	+	+	+	−	−	−	−	30	0.58	43	0.65
Dusky turtle dove	*Streptopelia lugens*	+	+	+	+	+	+	+	+	41	0.79	49	0.74
Lemon dove	*Columba larvata*	+	+	−	−	−	−	−	−	6	0.12	9	0.14
Yellow-fronted parrot	*Poicephalus flavifrons*	+	+	−	−	−	−	−	−	12	0.23	27	0.41
Black-winged lovebird	*Agapornis taranta*	+	+	+	+	+	+	−	−	79	1.5	118	1.78
White-cheeked turaco	*Menelikornis leucotis*	+	+	−	−	−	−	−	−	18	0.35	42	0.63
Verreaux's eagle-owl	*Ketupa lactea*	+	+	−	−	−	−	−	−	6	0.12	14	0.21
Montane nightjar	*Caprimulgus poliocephalus*	+	+	+	+	+	+	−	−	20	0.38	24	0.36
Little swift	*Apus affinis*	−	+	+	+	+	+	−	−	33	0.64	49	0.74
Nyanza swift	*Apus niansae*	+	+	−	−	+	+	−	−	50	0.96	68	1.02
Speckled mousebird	*Colius striatus*	+	+	+	+	−	−	−	−	81	1.56	113	1.7
Pied kingfisher	*Ceryle rudis*	−	−	−	−	−	−	−	+	6	0.12	18	0.27
Gray-headed kingfisher	*Halcyon leucocephala*	−	−	−	−	−	−	+	+	11	0.21	34	0.51
Giant kingfisher	*Megaceryle maxima*	−	−	+	−	−	−	+	+	15	0.29	14	0.21
Little bee-eater	*Merops pusillus*	+	+	+	+	+	+	−	−	32	0.61	53	0.8
Blue-breasted bee-eater	*Merops variegatus*	+	+	+	+	+	+	−	−	35	0.67	39	0.59
Black-billed wood hoopoe	*Phoeniculus somaliensis*	+	+	−	−	−	−	−	−	8	0.15	11	0.17
African hoopoe	*Upupa africana*	+	+	−	−	−	−	−	−	4	0.08	8	0.12
Red-billed hornbill	*Tockus erythrorhynchus*	+	+	+	+	−	−	−	−	24	0.46	36	0.54
Hemprich's hornbill	*Lophoceros hemprichii*	+	+	+	+	−	−	−	−	59	1.13	78	1.18
African gray hornbill	*Lophoceros nasutus*	+	+	+	+	−	−	−	−	82	1.58	92	1.39
Silvery-cheeked hornbill	*Bycanistes brevis*	+	+	−	−	−	−	−	−	16	0.31	20	0.3
Black-billed barbet	*Lybius guifsobalito*	+	+	−	−	−	−	−	−	8	0.15	24	0.36
Red-fronted tinkerbird	*Pogoniulus pusillus*	+	+	−	−	−	−	−	−	13	0.25	32	0.48
Nubian woodpecker	*Campethera nubica*	+	+	−	−	−	−	−	−	24	0.46	42	0.63
Abyssinian woodpecker	*Dendropicos abyssinicus*	+	+	−	−	−	−	−	−	12	0.23	18	0.27
Erlanger's lark	*Calandrella blanfordi erlangeri*	−	−	+	+	−	−	−	−	4	0.08	8	0.12
Thekla's lark	*Galerida theklae*	−	−	+	+	−	−	−	+	6	0.12	13	0.21
Rock martin	*Galerida theklae*	−	−	+	−	+	+	−	−	44	0.85	68	1.02
Lesser striped swallow	*Cecropis abyssinica*	+	+	−	−	−	−	−	−	14	0.27	124	1.86
Mountain wagtail	*Motacilla clara*	−	−	+	+	−	−	+	+	12	0.23	23	0.35
Common bulbul	*Pycnonotus barbatus*	+	+	+	+	−	−	−	−	124	2.38	158	2.38
Rüppell's robin-chat	*Cossypha semirufa*	+	+	+	+	+	−	−	−	63	1.21	124	1.87
Red-breasted wheatear	*Oenanthe bottae*	+	+	+	+	+	+	−	−	20	0.38	32	0.48
African stonechat	*Saxicola torquatus*	−	−	+	+	−	−	−	+	9	0.17	20	0.3
Abyssinian black wheatear	*Oenanthe lugubris*	−	−	−	−	+	+	−	−	12	0.23	24	0.36
Moorland chat	*Pinarochroa sordida*	+	+	+	+	−	−	−	−	26	0.5	45	0.68
Mocking cliff chat	*Thamnolaea cinnamomeiventris*	−	+	−	−	+	+	−	−	24	0.46	46	0.69
Rüppell's black chat	*Myrmecocichla melaena*	+	+	−	+	+	+	−	−	52	1.1	96	1.45
Groundscraper thrush	*Turdus litsitsirupa*	−	+	−	−	+	+	−	−	8	0.15	23	0.35
Little rock thrush	*Monticola rufocinereus*	+	−	−	−	+	+	−	−	13	0.25	11	0.17
Mountain thrush	*Turdus plebejus*	+	+	+	+	−	−	−	−	84	1.6	133	2
Cinnamon bracken warbler	*Bradypterus cinnamomeus*	+	+	−	+	−	−	−	−	23	0.44	32	0.48
Gray-backed camaroptera	*Camaroptera brevicaudata*	+	+	+	+	−	−	−	−	65	1.25	88	1.33
Brown woodland warbler	*Phylloscopus umbrovirens*	+	+	−	−	−	−	−	−	43	0.83	68	1.02
Ethiopian cisticola	*Cisticola lugubris*	+	+	−	−	−	−	−	−	9	0.17	11	0.17
Tawny-flanked prinia	*Prinia subflava*	+	+	+	+	−	−	−	−	19	0.36	30	0.45
Abyssinian slaty flycatcher	*Melaenornis chocolatinus*	+	+	2	−	−	−	−	−	11	0.21	16	0.24
African dusky flycatcher	*Muscicapa adusta*	+	+	+	+	−	−	−	−	13	0.25	20	0.52
African paradise flycatcher	*Terpsiphone viridis*	+	+	+	+	−	−	−	−	53	1.02	36	0.54
White-rumped babbler	*Turdoides leucopygia*	+	+	+	+	−	−	−	+	110	2.1	132	1.99
Abyssinian catbird	*Sylvia galinieri*	+	+	−	−	−	−	−	−	19	0.36	28	0.42
White-backed black tit	*Melaniparus leuconotus*	+	+	−	−	−	−	−	−	14	0.27	29	0.44
Montane white-eye	*Zosterops poliogastrus*	+	+	−	−	−	−	−	−	24	0.46	42	0.63
Tacazze sunbird	*Nectarinia tacazze*	+	+	+	+	−	−	+	+	56	1.08	56	0.84
Malachite sunbird	*Nectarinia famosa*	+	+	+	+	−	−	−	+	34	0.65	30	0.45
Scarlet-chested sunbird	*Chalcomitra senegalensis*	+	+	+	+	−	−	−	+	72	1.38	79	1.19
Variable sunbird	*Cinnyris venustus*	+	+	+	+	−	−	+	+	53	1.02	45	0.68
Common fiscal	*Lanius collaris*	+	+	+	+	−	−	−	−	24	0.46	35	0.53
Gray-backed fiscal	*Lanius excubitoroides*	+	+	+	+	−	−	+	+	20	0.38	28	0.42
Ethiopian boubou	*Laniarius aethiopicus*	+	+	−	−	−	−	−	−	86	1.65	112	1.69
Abyssinian oriole	*Oriolus monacha*	+	+	−	−	−	−	−	−	46	0.88	68	1.02
Fork-tailed drongo	*Dicrurus adsimilis*	+	+	−	−	−	−	−	−	24	0.46	43	0.65
Pied crow	*Corvus albus*	+	+	+	+	+	−	−	−	72	1.38	34	0.51
Cape rook	*Corvus capensis*	+	−	+	+	+	−	−	+	51	0.98	42	0.64
Fan-tailed raven	*Corvus rhipidurus*	−	+	+	+	+	+	−	+	42	0.81	68	1.02
Thick-billed raven	*Corvus crassirostris*	−	+	+	−	−	+	+	−	17	0.33	32	0.48
Red-billed oxpecker	*Buphagus erythrorynchus*	−	+	+	+	−	−	−	−	26	0.5	26	0.39
Red-winged starling	*Onychognathus morio*	+	+	+	+	+	−	+	+	136	2.6	190	2.86
Slender-billed starling	*Onychognathus tenuirostris*	+	+	+	+	+	+	−	−	48	0.92	39	0.59
Greater blue-eared starling	*Lamprotornis chalybaeus*	−	−	+	+	−	−	+	+	50	0.96	54	0.81
Swainson's sparrow	*Passer swainsonii*	+	+	+	+	−	−	−	+	71	1.36	139	2.1
Village weaver	*Ploceus cucullatus*	+	+	+	+	−	−	+	+	156	3.1	94	1.4
Spectacled weaver	*Ploceus ocularis*	+	+	+	+	−	−	+	+	129	2.48	103	1.6
Little weaver	*Ploceus luteolus*	+	+	+	+	−	−	+	+	111	2.13	84	1.3
Baglafecht weaver	*Ploceus baglafecht*	+	+	+	+	−	−	+	+	198	3.8	100	1.5
Yellow-crowned bishop	*Euplectes afer*	+	+	+	+	−	−	+	+	223	4.28	113	1.7
Yellow bishop	*Euplectes capensis*	+	+	+	+	−	−	+	+	207	3.98	92	1.4
Yellow-mantled widowbird	*Euplectes macroura*	+	−	+	+	−	−	−	−	50	0.96	19	0.29
Red-collared widowbird	*Euplectes ardens*	−	−	+	+	−	−	−	+	38	0.73	28	0.42
Red-cheeked cordon-bleu	*Uraeginthus bengalus*	+	+	+	+	+	−	+	+	44	0.85	94	1.4
Red-billed firefinch	*Lagonosticta senegala*	+	+	+	+	−	−	−	+	30	0.58	68	1.02
African firefinch	*Lagonosticta rubricata*	−	+	+	+	−	−	−	+	12	0.23	38	0.57
Yellow-bellied waxbill	*Coccopygia quartinia*	+	+	−	−	−	−	−	+	17	0.33	36	0.54
Common waxbill	*Estrilda astrild*	+	+	−	−	−	−	−	−	9	0.17	19	0.29
Pin-tailed whydah	*Vidua macroura*	+	+	+	+	−	−	−	+	36	0.69	19	0.29
Village indigobird	*Vidua chalybeata*	+	+	+	−	−	−	−	+	28	0.54	12	0.18
Yellow-crowned canary	*Serinus flavivertex*	+	+	+	+	−	−	−	+	55	1.06	27	0.41
African citril	*Crithagra citrinelloides*	+	+	+	+	−	−	−	+	57	1.09	49	0.74
Brown-rumped seedeater	*Crithagra tristriata*	+	+	+	+	+	−	+	+	101	1.94	162	2.4
Streaky seedeater	*Crithagra striolata*	+	+	+	+	−	−	+	+	45	0.86	95	1.4
Cinnamon-breasted bunting	*Emberiza tahapisi*	+	+	−	−	−	−	−	−	18	0.35	26	0.39

*Note:* +, present; −, absent.

Abbreviations: CR, critically endangered; Dry, dry season; E, endemic; NE, near endemic (endemic to Ethiopia and Eritrea); RA, relative abundance; Wet, wet season.

## Data Availability

Data is available on request from the corresponding author.
